# An optimal control framework for dynamic induction control of wind farms and their interaction with the atmospheric boundary layer

**DOI:** 10.1098/rsta.2016.0100

**Published:** 2017-03-06

**Authors:** W. Munters, J. Meyers

**Affiliations:** Department of Mechanical Engineering, KU Leuven, Celestijnenlaan 300A, 3001 Heverlee, Belgium

**Keywords:** wind farms, large-eddy simulations, optimal control

## Abstract

Complex turbine wake interactions play an important role in overall energy extraction in large wind farms. Current control strategies optimize individual turbine power, and lead to significant energy losses in wind farms compared with lone-standing wind turbines. In recent work, an optimal coordinated control framework was introduced (Goit & Meyers 2015 *J. Fluid Mech.* **768**, 5–50 (doi:10.1017/jfm.2015.70)). Here, we further elaborate on this framework, quantify the influence of optimization parameters and introduce new simulation results for which gains in power production of up to 21% are observed.

This article is part of the themed issue ‘Wind energy in complex terrains’.

## 1. Introduction

In large-scale wind farms, a large number of wind turbines are clustered together, and complex turbine wake interactions play an important role in energy extraction from the atmospheric boundary layer (ABL). Current control strategies typically maximize power extraction at turbine level, but do not take into account wind-farm wake interactions, leading to power deficits in downstream regions of the wind farm. More specifically, efficiency losses of up to 40% compared with lone-standing turbines have been reported in operational offshore wind farms [1,2]. Recently, Goit & Meyers [3] introduced an optimal coordinated control framework for dynamic induction control of wind-farm boundary layers for maximal energy extraction. In this framework, wind turbines are used as active flow actuators that optimally influence turbine wake interactions as well as the interplay of the wind farm with the turbulent ABL. To this end, a model-predictive optimal control approach was employed, in which the wind-farm boundary layer is modelled using large-eddy simulations (LES) of the Navier–Stokes partial differential equations (PDEs). As also explained in this paper, the method is not directly usable as a real controller, but instead provides benchmark results that yield insights into the potential of wind-farm control, which may help in elucidating new control mechanisms for wind farms. The framework was applied earlier to fully developed wind farms [3] as well as wind farms with entrance effects [4], for which gains in energy extraction of 16% and 7% were achieved, respectively.

Here, we review and further elaborate on the work introduced by Goit & Meyers [3]. We quantify the influence of optimization parameters, and reveal differences with previous studies. In addition, we present new results, further illustrating the potential of optimal control methods applied to wind-farm boundary layers. The paper is structured as follows: §2 introduces key aspects of the methodological approach. Section 3 introduces recent results, and quantifies the sensitivity of the performance of the overall optimal control framework to user-defined parameters. Finally, §4 provides a concise summary and indicates directions for future research.

## 2. Methodology

We discuss the aforementioned optimal control methodology, highlighting key aspects of and differences with previous work [3,4]. First, our receding horizon optimal control approach is discussed, and compared with classical model-predictive control methods. Subsequently, the PDE-constrained optimization problem is introduced. Afterwards, the actuator disc wind turbine model and the adjoint gradient calculation are discussed. Finally, an updated optimization algorithm is presented, that is based on the L-BFGS-B quasi-Newton method.

### (a) Receding horizon optimization

**Figure 1.**
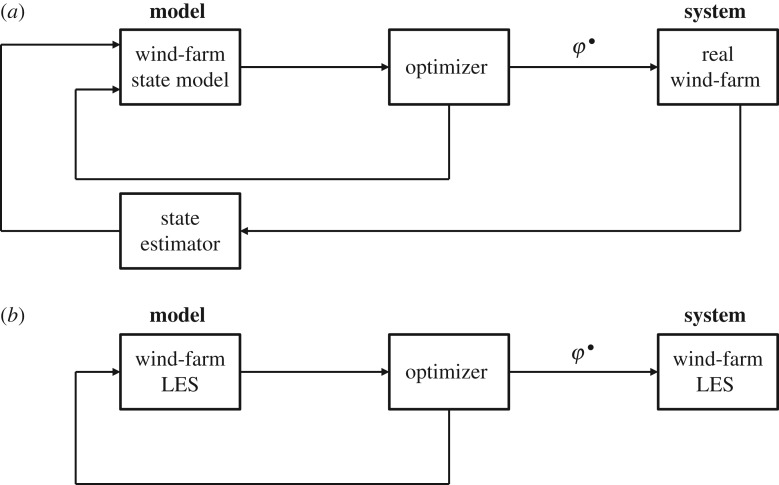
Model-predictive control applied to wind farms. (*a*) Conventional feedback model-predictive control of a wind farm. (*b*) Benchmark optimal control framework of a wind-farm LES considered in the current study.

**Figure 2.**
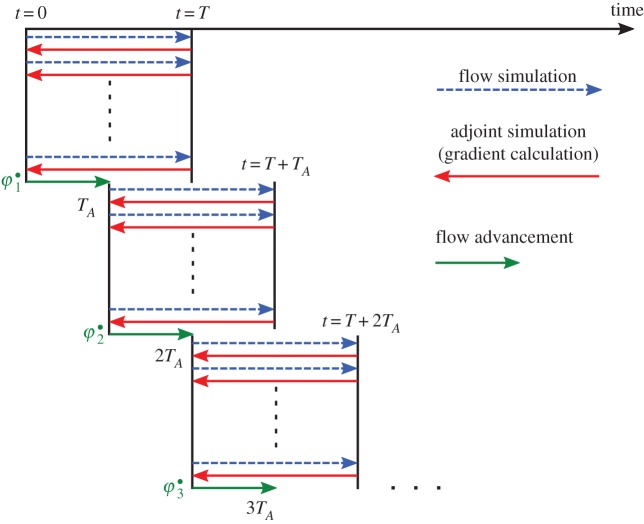
Receding horizon optimization framework for optimal control of wind farms. Figure adapted from ref. [3]. (Online version in colour.)

In a conventional optimal control approach (figure 1*a*), the system (the wind farm) is controlled by a controller that optimizes the controls (***φ***) in a state model that represents the system. Usually, a receding horizon approach is used (e.g. figure 2), in which the controls are optimized for a future time window [*t*,*t*+*T*], after which the obtained optimum (***φ***^•^) is used during a control time step *T*_*A*_. In addition, the model state is regularly adapted based on measurements in the system, minimizing the errors between the state model and the real system. The challenge for wind farms is that the detailed turbulent flow state in the ABL is very high-dimensional, and an accurate state model (e.g. based on LES) is computationally very expensive, so that it is impossible to implement in a real-time controller. Therefore, wind-farm control in practice requires extensive simplifying assumptions that may limit *a priori* the performance of the controller. Because of this, the development of practical cooperative wind-farm controllers remains an open issue to date, in particular for the case of power maximization through mitigation of unfavourable wake interactions.

Instead of focusing on the direct formulation of a real wind-farm controller for increased energy extraction, Goit & Meyers [3] concentrated on finding optimal controls in a very accurate state representation of the wind-farm boundary layer, i.e. based on LES that resolve the large turbulent flow structures in the ABL and their interaction with the wind turbines both in space and in time (figure 1*b*). Such an approach is not yet feasible for real control, as LES is, to date, still significantly slower than real time, but it allows one to explore the potential of improved energy extraction by wind-farm control, and may help in identifying new ways in which large wind farms can optimally interact with the ABL.

In the current work, we further elaborate and adapt the framework of Goit & Meyers [3,4], using a receding horizon optimization approach as shown in figure 2. Given a control time horizon *T*, controls are optimized to yield maximum energy extraction. Further details are provided in §§2b–e. Part of the resulting optimal controls ***φ***^•^ are then used for a time period *T*_*A*_≤*T*, during which we advance the wind-farm LES system in time. Subsequently, control optimization over a new time horizon is initiated, etc. The flow advancement time *T*_*A*_ is chosen based on a trade-off between limiting overall computational expenses, favouring 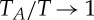, and averting finite-horizon optimization effects, favouring 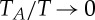. The influence of the ratio *T*_*A*_/*T* on the dynamic behaviour of the optimized wind farm is discussed in §3, presenting results for both *T*_*A*_=*T*/2 and *T*_*A*_=*T*/4, and evaluated over a total wind-farm control time *T*_tot_=*N*_*A*_*T*_*A*_ of approximately 30 min of real wind-farm time.

### (b) Definition of the optimization problem

Within each window of the receding horizon framework introduced above, we maximize aggregated energy extraction by solving the following PDE-constrained optimization problem:
2.1
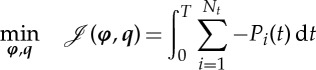

2.2
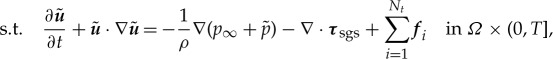

2.3
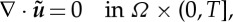

2.4
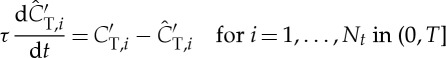

2.5
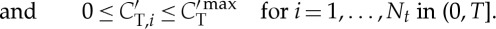

The cost functional to be minimized in (2.1) is the total wind-farm energy extraction from the boundary layer over a time horizon *T*, with switched sign for convention of notation. The control variables in the problem defined above are the time-dependent disc-based thrust coefficients for all turbines ***φ***≡[*C*′_T,1_(*t*),*C*′_T,2_(*t*),…,*C*′_T,*N*_*t*__(*t*)], whereas the state variables 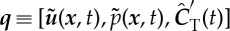 (where 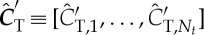) consist of the velocity and pressure field in the ABL, and time-filtered turbine thrust coefficients. The explicit dependence of both turbine power *P*_*i*_ and thrust force ***f***_*i*_ on the latter is discussed in the next section on turbine modelling. The optimization problem is solved in a reduced form, i.e. instead of explicitly considering the differential equations in (2.2)–(2.4) as constraints and optimizing over the parameter space [***φ***,***q***], the dependence of the state on the controls ***q***(***φ***) is satisfied explicitly through means of LES, leaving only the control parameter ***φ*** as decision variable to optimize the reduced cost functional 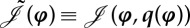.

**Figure 3.**
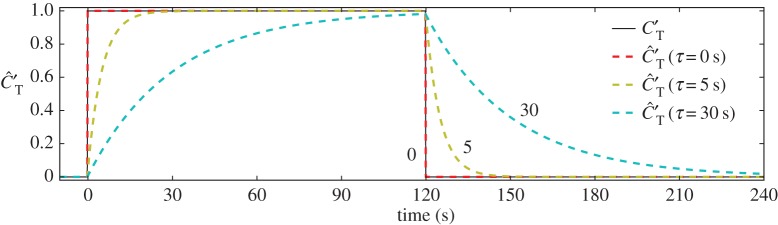
Illustration of the response of the thrust coefficient 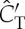 to a square-wave variation in the control signal *C*_T_′ for varying wind-turbine response times *τ*. (Online version in colour.)

The filtered Navier–Stokes state equations in (2.2) and (2.3) are solved using an in-house LES solver [3,5,6]. The equations are discretized using a Fourier pseudo-spectral approach in the horizontal directions, combined with a fourth-order finite-difference scheme in the vertical direction [7]. The top surface is treated using a free-slip condition, whereas the bottom surface applies a wall stress boundary condition. The influence of Coriolis forces and thermal stratification is not taken into account, i.e. all cases considered here involve half-channel flows serving as a surrogate for a neutral ABL. The high Reynolds numbers associated with the latter justify neglecting resolved effects of molecular viscosity. Instead, the effect of subgrid-scale (sgs) phenomena on resolved scales is represented using a classical Smagorinsky model with a constant coefficient *C*_*s*_=0.14, in which the Smagorinsky length scale is reduced near the surface using a wall damping function [8]. Inflow conditions are extracted from an auxiliary periodic precursor simulation and are imposed using a fringe region [9]. The flow field is advanced in time using an explicit fourth-order Runge–Kutta scheme using a constant time step corresponding to a Courant–Friedrichs–Lewy number of 0.4. The time-filtering state equation (2.4) applies a first-order exponential time filter to the control input *C*_T_′ to generate the thrust coefficient 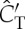 that is used in the thrust force felt by the flow. An implicit Euler scheme is used to integrate the time-filtering equation. To give an indication of the influence of the wind-turbine response time *τ*, defined as the filter time constant, on the thrust coefficient behaviour, the response of the latter to a square-wave variation in the control signal is illustrated in figure 3. Note, however, that the control signals originating from the optimal control simulations will be more complicated. In this way, (2.4) allows one to incorporate a finite wind-turbine response time *τ* to temporal variations in optimized thrust coefficient setpoints *C*_T_′. This would, for example, fit in a hierarchical approach to wind-farm control, in which wind-farm control signals are passed on to individual turbine controllers that themselves may not react instantaneously to these control signals. In the current work, we use different time constants *τ* to investigate the effect of control smoothness on possible power gains. Finally, the box constraints (2.5) on the controls are added that prevent the turbine from operating as a fan on the one hand, and limit the maximal thrust coefficient to technically feasible values on the other hand (see below).

### (c) Wind-turbine modelling

The forces exerted by turbine *i* on the boundary layer flow are parametrized using a non-rotating actuator disc method as
2.6
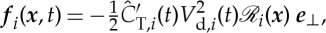

where 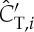 is the time-filtered disc-based thrust coefficient (e.g. [5,6]), ***e***_⊥_ is the unit vector perpendicular to the rotor plane, and 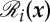 is a smoothed representation of the geometric footprint of the rotor on the LES grid. Furthermore, the disc-averaged velocity is calculated as 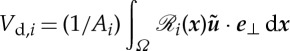, with *A*_*i*_ the rotor disc area. Note that, through the time-filtering equation (2.4), the actual time-filtered thrust coefficient 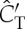 employed by the turbine can be rendered arbitrarily smooth in time, which allows one to model a finite wind-turbine response time. This is in contrast to previous studies [3,5,6], where turbine inertia was modelled through time filtering of the disc velocity *V*_d,*i*_. The actual mechanical power captured by the wind turbine is calculated as
2.7
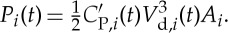

In contrast to earlier work [3,5,6], where 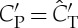, here the disc-based power coefficient 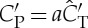, with *a*=0.9 for the simulation grids applied in this paper. This relation is derived from a fitting operation of LES results to momentum theory as described in appendix A. This is done in order to eliminate overpredictions in power extraction observed using turbine parametrizations on typical wind-farm grid resolutions (e.g. [10,11]). Owing to the linear nature of this fit, this does not influence the relative gains in power production discussed below, which are normalized by a reference case.

Following the methodology in Goit & Meyers [3], dynamic induction control is performed by controlling the disc-based thrust coefficient *C*_T_′ for every turbine and in every time step, such that the aggregated power extraction of the entire wind farm is maximized. In the remainder of this section, we discuss steady-state turbine operation, hence *C*_T_′ and 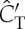 can be used interchangeably. Using relations from one-dimensional (1D) momentum theory for an ideal turbine [12], straightforward algebraic manipulation relates the disc-based thrust coefficient *C*_T_′ with the conventional power coefficient *C*_P_ and thrust coefficient *C*_T_ as
2.8
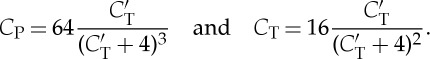

**Figure 4.**
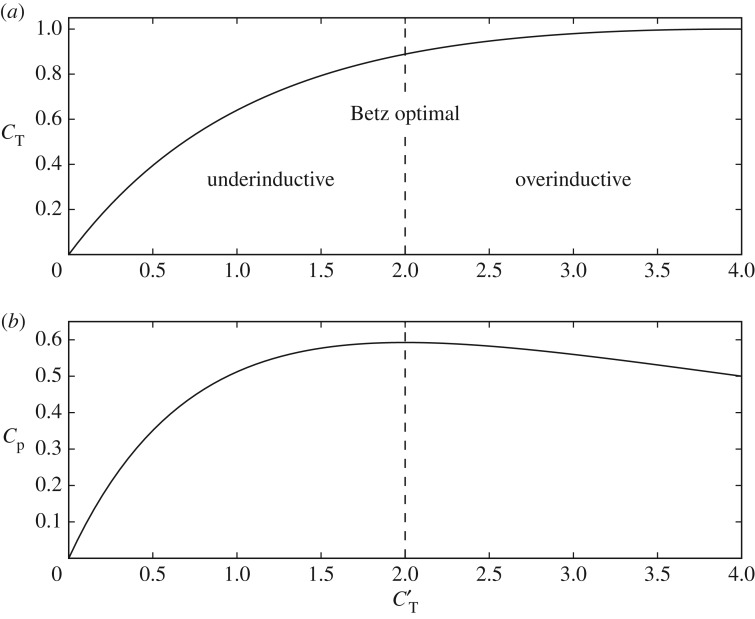
Thrust coefficient *C*_T_ (*a*) and power coefficient *C*_P_ (*b*) as a function of modified thrust coefficient *C*_T_′ for a steady turbine based on one-dimensional momentum theory.

As shown in figure 4, *C*_P_ achieves a maximum at the well-known Betz limit of 
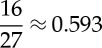
, for which *C*_T_′=2. In order to obtain any lower *C*_P_ value, two possible *C*_T_′ setpoints can be chosen: an underinductive (*C*_T_′<2) and an overinductive (*C*_T_′>2) coefficient. In the work of Goit & Meyers [3], a maximum coefficient 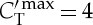 is used. It can be seen from figure 4 that this corresponds to a maximal thrust force, with *C*_T_=1. Furthermore, it is worth noting that *C*_P_ is quite insensitive to *C*_T_′ in the range 1.5–2.5, indicating that, in this zone, turbine thrust forces can be varied significantly without sacrificing a lot of power.

### (d) Gradient calculation

Owing to the combination of a high control space dimensionality (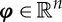, with *n*≈10^4^–10^5^) with a computationally expensive system of state equations, calculating the gradient of the reduced cost functional 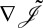 using classical finite-difference methods is computationally infeasible. Instead, we employ the continuous adjoint method, in which the gradient is identified through the solution of an additional set of PDEs. The simulation of these adjoint equations involves a computational cost similar to a single state system evaluation, independent of control space dimensionality *n*. The adjoint equations are derived by appending the state constraints to the cost function in (2.1)–(2.4) to form the Lagrangian, introducing the triplet of adjoint variables q*=[***ξ***, *π*, ***σ***] as the Lagrange multipliers to the state variables 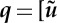, 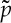, 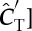. Postulating vanishing gradients of the Lagrangian with respect to the state variables directly produces the adjoint equations [3,13,14]:
2.9
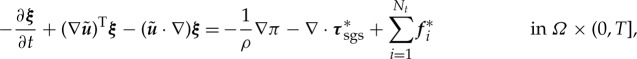

2.10
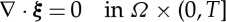

2.11
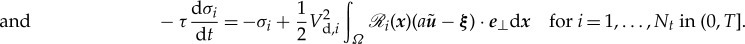

The definition and derivation of the adjoint equations is identical to the previous work [3,4]. However, due to the addition of a time-filtering equation on the controls and the resulting definition of the turbine forces, the adjoint forcing terms 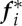 associated with each of the turbines differ from the latter studies, and are given by
2.12
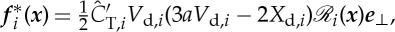

where 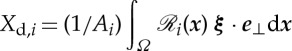 is the disc-averaged adjoint velocity, defined similarly to *V*_d,*i*_. The gradient can then be evaluated as
2.13
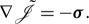

The derivation of the adjoint time-filtering equation and the novel expressions for both the adjoint forcing terms and the cost functional gradient are presented in appendix B.

### (e) Optimization algorithm

In this work, we solve the optimal control problem within each prediction window using the quasi-Newton L-BFGS-B algorithm by Byrd *et al.* [15]. This is in contrast to the classical use of nonlinear conjugate gradient (CG) methods in flow control, for instance, in earlier studies on drag reduction [14], noise reduction [16] and wind farms [3,4]. First, the main steps in the algorithm are briefly outlined. Afterwards, the convergence behaviour of the method is compared with a nonlinear CG method for a typical wind-farm control case.

At the beginning of each iteration, a quadratic model of the cost functional *m*_*k*_(***φ***) is constructed based on the current iterate 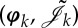, the current gradient 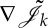 and a positive definite limited-memory Hessian approximation *B*_*k*_ as
2.14
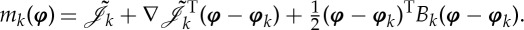

As discussed in [15], this model is minimized while satisfying the box constraints (i.e. 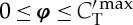), yielding a solution 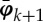. Subsequently, a Moré–Thuente line search [17] along direction 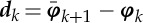 is employed to identify the new iterate ***φ***_*k*+1_=***φ***_*k*_+*α*_*k*_***d***_*k*_, such that the strong Wolfe conditions are satisfied [18,19]. The line search is initialized with a unit step length *α*_*k*_=1, which, as shown below, often directly meets the Wolfe conditions. Note that each iteration requires a minimum of one LES and one adjoint simulation, since the construction of *m*_*k*_ requires both the current functional value 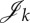 and its gradient 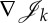. In addition, checking the Wolfe conditions entails an additional pair of LES and adjoint evaluations per inner line search iteration. The limited-memory Hessian approximation *B*_*k*_ is constructed from information retained from previous iterations through a set of *m* correction pairs 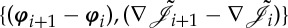, for *i*=*k*−1,…,*k*−*m* [20].

**Figure 5.**
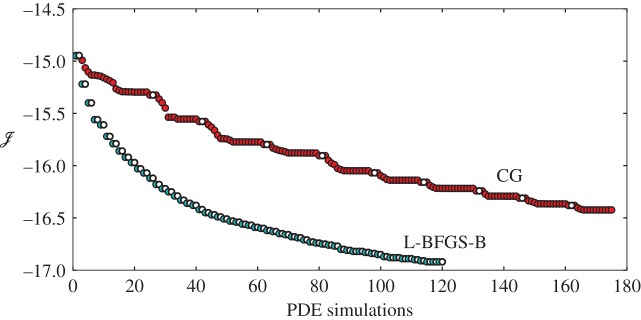
Cost functional decrease as a function of PDE simulations for a nonlinear CG and L-BFGS-B algorithm applied to a typical wind-farm optimization window. Filled circles, LES; open circles, adjoint. (Online version in colour.)

Figure 5 presents the cost function decrease for a typical wind-farm optimization window as a function of PDE evaluations for the Polak–Ribière nonlinear CG method and the L-BFGS-B method. Figure 5 illustrates that the use of additional knowledge of curvature information employed by the L-BFGS-B algorithm significantly speeds up convergence compared with CG. Recently, this behaviour was also shown for a low-Reynolds-number turbulent channel flow case [21]. Note that, for PDE-constrained optimization, the most important driver for computational costs is, by far, the amount of PDE evaluations. Therefore, it can be seen from Figure 5 that, for a given decrease in the cost functional, computational efforts are reduced by a factor 4 for L-BFGS-B compared with CG. The convergence rate of the optimization algorithm and its sensitivity to user-defined parameters are further discussed based on a set of recent optimal control cases in §3. In addition to the updated optimization algorithm, a further decrease in time to solution of the optimal control simulations is achieved by the upgrade of the grid partitioning scheme used in the code parallelization from a 1D slab decomposition to a two-dimensional pencil decomposition [22,23], which allows the PDE solver to scale to core counts in the order of a hundred to a thousand cores for the simulation grids considered here.

### (f) Simulation set-up

In the remainder of the paper, we show new results of optimized wind farms. We focus mainly on the justification of methodological choices and parameter settings, with the aim of quantifying the influence of optimization parameters on convergence rate and value of the optimized cost functional for the optimal control framework described in this paper.

**Figure 6.**
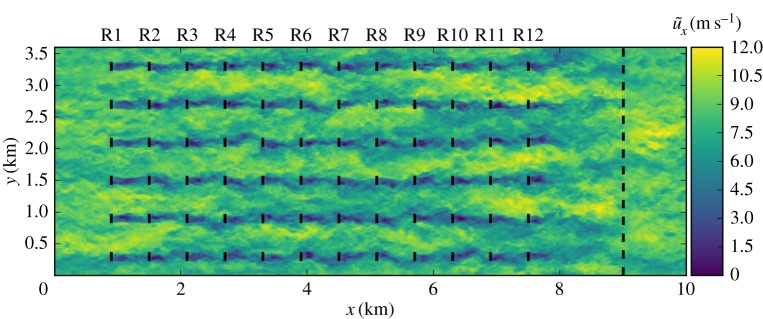
LES streamwise velocity field for a 12×6 aligned wind farm. The black lines represent the turbine rotors. The black dashed line indicates a buffer region for the imposition of inflow conditions [9,4]. (Online version in colour.)

We consider a 12×6 aligned wind farm where turbines are spaced apart by six rotor diameters in axial and transverse directions. The spatial domain of 10×3.6×1 km^3^ is discretized using a standard numerical grid of 384×192×244. A driving pressure gradient of 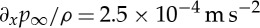 results in a free-stream hub-height velocity slightly over 8 m s^−1^. Figure 6 illustrates a typical snapshot of the LES velocity field for the wind farm considered here. It can be seen from figure 6 that, starting from the second row, turbines are subjected to significantly lower velocities with increased variability. Moreover, it is shown that the LES allows one to capture complex unsteady wake behaviour, including meandering and turbulent entrainment, that can potentially be harnessed by the optimal control approach. Unsteady turbulent inflow conditions are the same for all cases, and are generated by running a separate precursor simulation of a periodic ABL without turbines on a domain identical to that of the wind-farm simulation. Wind-farm operation is optimized over *N*_*A*_=15 prediction windows, resulting in a total time of *T*_tot_=*N*_*A*_*T*_*A*_=30 min. Time integration is performed using a constant time step Δ*t*=0.75 s. We employ a prediction horizon of *T*=240 s, and a flow advancement horizon of *T*_*A*_=*T*/2=120 s. Moreover, we retain *m*=5 correction pairs for the BFGS Hessian updates, and stop the optimization algorithm after *N*_it_=60 iterations. The chosen values for these additional optimization parameters are justified based on parameter studies discussed below.

## 3. Results and discussion

In this section, we discuss optimization results using the set-up mentioned above. First, the different cases are distinguished. Subsequently, the energy gains in optimized cases are quantified and discussed. Finally, the influence of optimization parameters is quantified.

**Figure 7.**
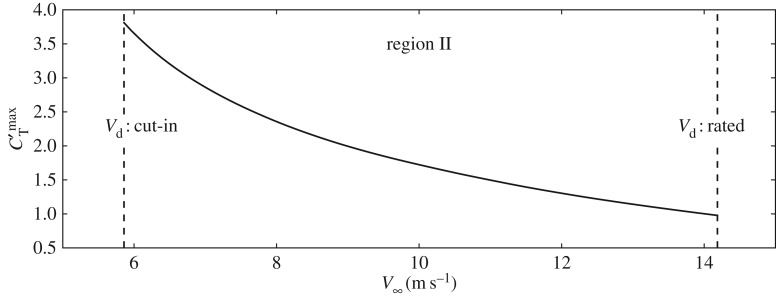
Maximum attainable modified thrust coefficient 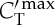 for region II operation of the NREL 5 MW turbine through increase of *λ* as a function of undisturbed upstream velocity 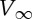.

We define a reference case R, in which all turbines are controlled statically and greedily, i.e. with thrust coefficients equal to the Betz-optimal value *C*_T_′=2. In addition, we define a total of six optimal control cases. The latter are distinguished by whether or not overinduction is allowed, i.e. with 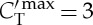 or 2, respectively, and by the wind-turbine response time *τ* of 0, 5 or 30 s. Optimized cases are denoted by *C*〈*X*〉*t*〈*Y* 〉, where *X* and *Y* represent 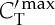 and *τ*, respectively, e.g. C3t30 for the case with 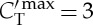 and *τ*=30 s. The upper bound 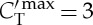 of the overinductive cases is justified based on figure 7, which contains results of blade element momentum theory calculations for the maximum attainable *C*_T_′ in the NREL 5 MW turbine [24] for region II operation through the increase of the tip speed ratio 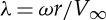, with *r* the turbine radius, *ω* its rotational speed and 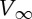 the undisturbed upstream velocity. The calculations are performed for a turbine in region II operation at below rated wind speeds, in which *λ* is controlled through regulation of the turbine generator torque. It can be seen that in this way, for 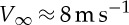, a *C*_T_′ of approximately 2.5 is feasible. This value could still be increased, e.g. through a minor increase in blade chord length. Therefore, the upper bound of 3 for the overinductive cases in this work is reasonable.

**Figure 8.**
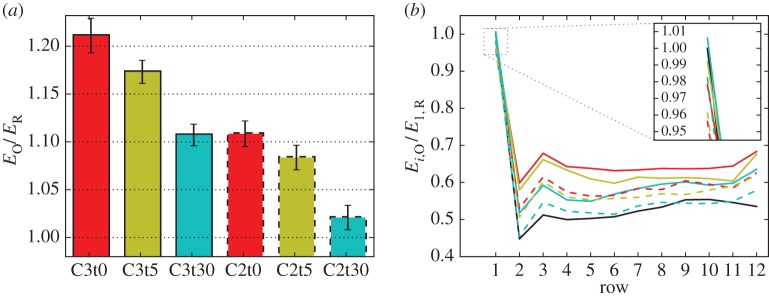
Energyextraction of optimally controlled wind farms *E*_O_ normalized by greedy reference case *E*_*R*_. (*a*) Total energy extraction. Error bars indicate confidence intervals of ±2 standard deviations. (*b*) Energy extraction per row, normalized by first row of reference case. From top: C3t0, red line; C3t5, yellow line; C3t30, light-blue line; C2t0, red dashed line; C2t5, yellow dashed line; C2t30, light-blue dashed line; R, black line. (Online version in colour.)

Figure 8 depicts the energy extraction of the optimally controlled wind farms normalized by the reference case. The energy extraction is integrated starting from the second optimization window, since the optimized wind farms are still in a transient phase during the first window. It is shown in figure 8*a* that all cases, except for the most restrictive case C2t30, achieve significant energy gains ranging between 8% and 21%. This illustrates the potential of dynamic coordinated control approaches over greedy individual control. As can be expected, decreasing flexibility with regard to 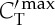 and *τ* reduces the margins for increased power extraction. It is worth noting that the slow-response overinductive case C3t30 achieves power gains close to the underinductive fast-response case C2t0 and C2t5. This observation is promising for the eventual actual roll-out of dynamic wind-farm controllers, as it shows that fast temporal variations in turbine control settings, with associated detrimental effects on turbine structural loading, are not a prerequisite for significant improvements in power extraction. The error bars in figure 8 indicate two standard deviations from the sampled mean. Error estimates are within ±1.2% points for all cases except C3t0, for which ±1.9% points are observed. The variances of relative energy gains are estimated using variances and covariances defined on the optimization window level, as further elaborated in appendix C. Note that the cases considered here feature significantly larger energy gains than the comparable case of Goit *et al.* [4], for which an increase in energy extraction of approximately 6% was reported. This fact can be attributed to a better convergence of the current results due to a judicious choice of optimization parameters as elaborated below and the update to a quasi-Newton optimizer discussed previously. In figure 8*b*, it is shown that energy extraction is increased in every downstream row by slightly downrating the first row for all cases except C3t30. Moreover, the first row aside, the last row extracts the most energy for all cases, since it does not have to take into account any downstream neighbours. We further performed for the overinductive case with *τ*=0 s a case in which only the first row turbines were optimally controlled (details not further shown here). We found energy gains of only 5% instead of 21%, indicating that all turbines contribute significantly to the energy gains.

**Figure 9.**
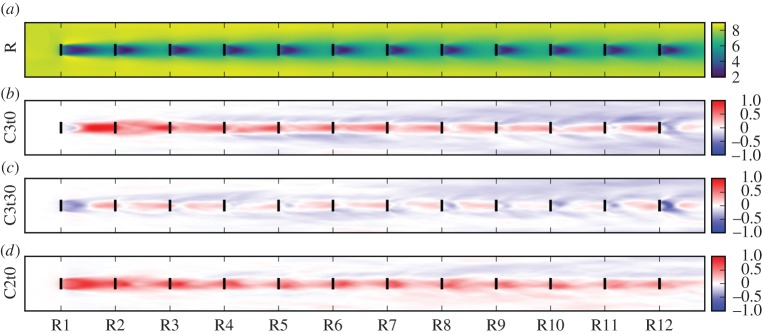
Plan views of time-averaged axial velocity 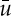 throughout the wind farm, averaged over all six turbine columns. (*a*) Reference case 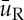. (*b*)–(*d*) Differences 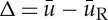 between optimized cases and reference case for C3t0, C3t30 and C2t0, respectively. (Online version in colour.)

Figure 9 shows time-averaged axial velocities throughout the wind farm. Figure 9*a* illustrates the axial velocity for the reference case. It can be seen that downstream turbines are subjected to the wakes of their upstream neighbours, and that wake behaviour, i.e. spanwise wake extent and wake recovery, achieves a fully developed regime in the downstream rows of the wind farm. Figure 9*b*–*d* depicts the difference between axial velocities in the optimized case and the reference case for cases C3t0, C3t30 and C2t0, respectively. It is shown that, for all cases, rows 2–12 are subjected to higher incoming velocities. For the overinductive cases C3t0 and C3t30, it can be seen that the near wake behind some turbines is deeper, yet the wake recovers more than in the reference case before reaching the next row. This is caused by enhanced turbulent wake mixing, consistent with observed increased turbulence intensities for downstream rows in these cases (not shown here). Contrastingly, the underinductive case C2t0 features higher axial velocities throughout the entire wind farm. Considering the comparable power gains of 10% for C3t30 and C2t0, this indicates that different cases are characterized by complex different mechanisms for power increase. These are not further elaborated here, and are the subject of current work. Ongoing research focuses on identifying the physical mechanisms behind the increase in energy extraction and the identification of possible correlations between turbines.

**Figure 10.**
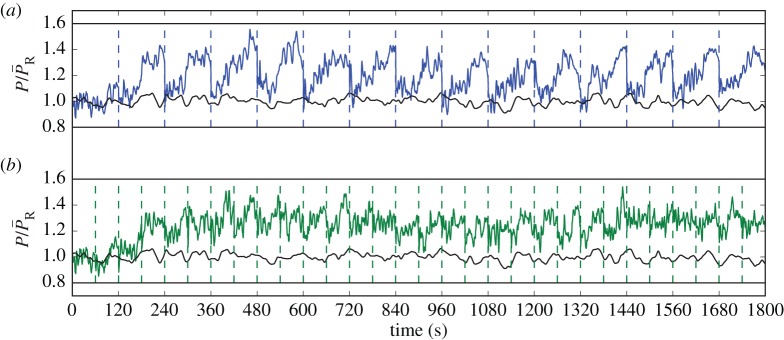
Dynamicbehaviour of optimized power extraction normalized by a greedy control reference case (smoother line, shown in black): (*a*) *T*_*A*_=*T*/2= 120 s; (*b*) *T*_*A*_=*T*/4=60 s. The dashed vertical lines indicate the flow advancement windows. (Online version in colour.)

Figure 10 qualitatively illustrates the influence of *T*_*A*_ on the temporal smoothness of the wind-farm power extraction for case C3t0. Figure 10 shows the normalized power extraction for *T*_*A*_=*T*/2=120 s (*a*) and *T*_*A*_=*T*/4=60 s (*b*), where both simulations apply an equal prediction horizon *T*=240 s. For *T*_*A*_=*T*/2, it can be seen in figure 10 that, within each window, power extraction is initially reduced. This causes flow conditions that lead to an increased power extraction later in the window. By stringing the windows together, a sawtooth-like behaviour of power extraction is created. Reducing *T*_*A*_ diminishes this effect, as shown in figure 10*b*, but leads to a doubling in computational cost for a given total time *T*_tot_. Although present-day computational resources have limited us to using *T*_*A*_=*T*/2=240 s for the cases presented in this paper, we aim to reduce *T*_*A*_/*T* in the future to obtain reduced ramps in power production and mitigate finite-horizon effects.

**Figure 11.**
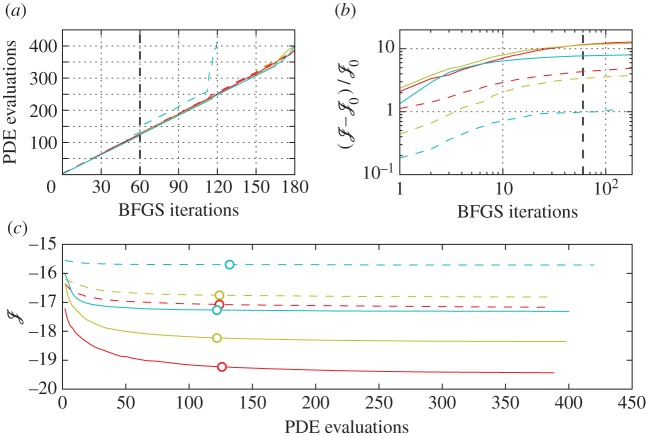
Typicalconvergence behaviour within an optimization window: C3t0, red line; C3t5, yellow line; C3t30, light-blue line; C2t0, red dashed line; C2t5, yellow dashed line; C2t30, light-blue dashed line (curves from bottom in (*a*) and (*c*), and from top in (*b*)). (Online version in colour.)

Given that limitations on computational resources prevent formal convergence of the optimal control problems considered here, we further investigate convergence rates, parameter sensitivities and the existence of local minima. Figure 11 illustrates the convergence behaviour for all optimal control simulations. From figure 11*a*, it can be seen that, for most cases, the amount of PDE evaluations is only slightly higher than twice the amount of BFGS iterations *N*_*it*_, illustrating that the optimization algorithm mostly takes steps with unit length, and only rarely has to resort to a line search when *α* does not satisfy the Wolfe conditions. Note that case C2t30 achieved convergence after 120 iterations, with a large increase in PDE simulations in the preceding iterations. This indicates that, upon approaching convergence, inconsistencies between the gradient obtained from the continuous adjoint approach and the actual cost functional gradient are making it harder to satisfy the Wolfe conditions, and more line searches are needed. This behaviour also starts to show in the later iterations of the other cases. Figure 11*b* depicts the relative improvement in the cost function within an optimization window in terms of the amount of iterations. It can be seen that, after 60 iterations, the improvement rate of the cost function significantly diminishes. Therefore, in order to limit computational expenses, in practice we stop all optimizations after *N*_it_=60. Figure 11*c* shows the cost functional as a function of PDE evaluations. The circle markers indicate the data points for which *N*_it_=60. Figure 11*c* shows that all cases achieve reasonable convergence for the chosen iteration limit, i.e. further improvements of the cost functional are minimal.

**Figure 12.**
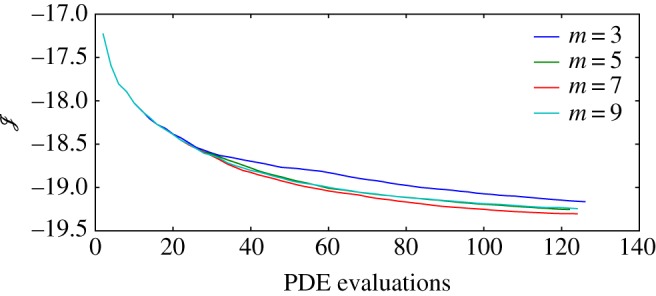
Cost functional as a function of PDE evaluations within an optimization for varying amount *m* of retained correction pairs for Hessian approximation. (Online version in colour.)

Figure 12 shows the sensitivity of convergence rate of C3t0 to *m*, i.e. the amount of retained correction pairs used in the update for the Hessian approximation in the L-BFGS-B algorithm. It can be seen from Figure 12 that *m*=3 converges more slowly than *m*=5, 7 or 9. The fact that using more Hessian correction pairs, i.e. *m*=9 over *m*=7, does not always lead to an improvement in convergence rate can be attributed to the irrelevance of gradient information from many iterations earlier. In the remainder of this work, we choose *m*=5, as this proved to be an adequate parameter choice, both here and for a varied set of constrained problems in the CUTE collection [25], as illustrated by Byrd *et al.* [15].

**Figure 13.**
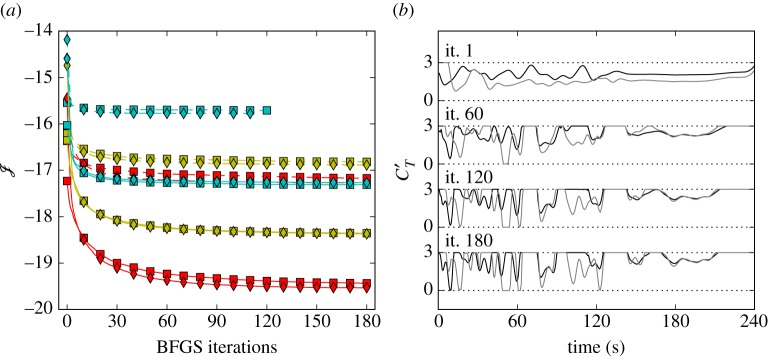
Sensitivityof optimization to initial conditions. (*a*) Cost functional as a function of BFGS iterations for a single window: squares, ***φ***_0_=2; diamonds, ***φ***_0_=1.33; C3t0, red line; C3t5, yellow line; C3t30, blue line; C2t0, red dashed line; C2t5, yellow dashed line; C2t30, light-blue dashed line (from bottom to top). (*b*) Optimized controls for a front row turbine at various iterations (black, ***φ***_0_=2; grey, ***φ***_0_=1.33). (Online version in colour.)

Given the non-convexity of the Navier–Stokes constrained optimization problems considered here, the cost functional landscape is expected to be characterized by a multitude of local optima. Figure 13 shows the cost functional and a subset of the optimized controls for different starting points ***φ***_0_, i.e. a greedy control case on the one hand, for which all turbines operate Betz-optimal at ***φ***_0_=[2, 2, …, 2], and a case with ***φ***_0_=[1.33, 1.33, …, 1.33] (e.g. [3,6]) on the other hand. Figure 13*a* illustrates cost functional decrease in terms of BFGS iterations. It can be seen that, although the choice of initial conditions has an influence on the power extraction, the relative order of which conditions yield better results is not uniform across all cases. Figure 13*b* shows the optimized controls for the first row of the wind farm after 1, 60, 120 and 180 iterations. Although some regions of the controls correspond between both initial conditions, mainly at the start of the optimization window, the controls differ significantly. Since the overall energy extraction seems only modestly dependent on the initial condition, the greedy control with *φ*_0_=2 is a suitable starting point for wind-farm optimal control.

In this section, new optimization results were introduced, illustrating the further potential of the optimal dynamic induction control framework introduced by Goit & Meyers [3]. Moreover, the choice of *T*_*A*_=*T*/2 is justified. The convergence behaviour of the optimization in terms of the amount of iterations and Hessian correction pairs *m* is illustrated. Finally, although the existence of local optima is shown, overall energy gains are found to be only slightly dependent on initial control parameters in the optimization problem.

## 4. Conclusion

In this work, we discuss and further elaborate on the PDE-constrained optimal control framework for dynamic induction control of wind farms introduced in Goit & Meyers [3]. Key components of the framework are discussed in detail, and the increase in convergence rate due to the upgrade of the optimization algorithm from a nonlinear CG method used in earlier work [3,4] to a quasi-Newton L-BFGS-B method is shown. New results indicate power gains for wind farms with optimal coordinated control in the order of 8–21% compared with a greedily controlled case. Simulation results show that fast variations in turbine thrust coefficients are not a prerequisite for significant gains in energy extraction. Finally, user-defined values of key parameters in the optimization approach are justified based on parameter studies. Future application of the optimization framework will focus on identifying the physical mechanisms behind the observed gains in energy extraction, and the translation of simulation-based optimal controllers with high computational costs towards practical cooperative wind-farm controllers. For instance, reduced-order state models, e.g. based on proper orthogonal decomposition or dynamic mode decomposition, provide a possible route to real-time implementation of optimal dynamic induction controllers. Another area of interest is the use of higher-fidelity wind turbine models, such as the rotating actuator disc model [26], the actuator sector model [27] or the actuator line model [28,29]. Furthermore, the addition of yaw to the optimal control problem and the inclusion of thermal stratification effects in the atmosphere are interesting topics for further research.

## Figures and Tables

**Figure 14.**
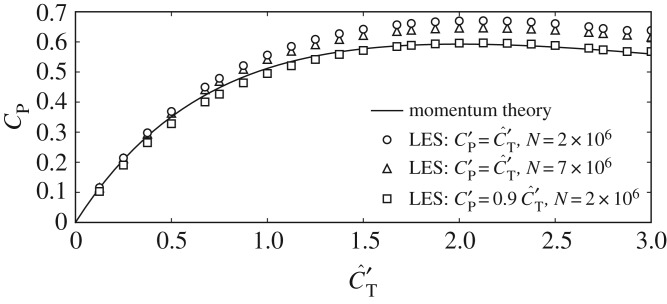
Power coefficient *C*_P_ as a function of time-filtered modified thrust coefficient 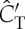 for a steady turbine in uniform inflow.

## Data accessibility

Datasets supporting this article can be found in a figshare repository [30]. These datasets include time-averaged flow fields, optimized controls and optimized wind-farm power extraction for all cases reported here.

## Appendix A. Relationship between modified power coefficient and modified thrust coefficient

Owing to present-day computational constraints, state-of-the-art wind-farm LES grids in pseudo-spectral codes are limited to ≈10 grid points across the turbine diameter. As illustrated below, actuator disc models for wind turbines in these resolutions lead to overpredictions in turbine power production (see e.g. refs [10,11]). Reviewing the expression for the disc-averaged velocity,

A 1

it can be shown that the main culprit in this overprediction is the geometric footprint , which is based on a Gaussian filter kernel , as discussed in more detail in [5,6]. We select the characteristic filter width as , with Δ the LES grid spacing. The diffuse smearing of the rotor geometry on the LES grid, which is required for numerical stability, leads to an overestimation of disc velocity *V*_d,*i*_, entailing an overprediction in power production compared with momentum theory. Note that this overprediction is not unique to actuator disc models; also in actuator line models overpredictions in turbine power are observed, for which a heuristic blade tip correction is often applied [10,11].

Figure 14 illustrates *C*_P_ as a function of , obtained from a steady-state actuator disc under uniform inflow. The circle markers indicate simulation results with  for the same resolution employed for the cases in §3, and illustrate an overpredicted *C*_P_. Consistent with the fact that the overprediction in power is caused by an overestimation in disc velocity due to the diffuse rotor representation, the error increases for increasing , since velocity gradients are higher for larger thrust forces. Although it is shown that this overprediction is diminished for higher grid resolutions (triangles), this is not a practical solution since present-day computational constraints do not allow optimal control simulations on finer grids. Therefore, a solution to this problem is to calibrate *C*_P_′ based on a least-squares fit to results obtained from 1D momentum theory, yielding , with *a*=0.9. From figure 14 it can be seen (squares) that this improves the *C*_P_ values significantly, and that the Betz limit is not surpassed. Physically, this implies that 10% of the power depleted from the boundary layer due to the turbine presence is not captured by the rotor, but can be ascribed to dissipation losses. We remark that the coefficient *a* depends on the simulation grid, and will tend to a value of 1 for finer and finer grids. It is worth noting that, since the proposed fit is linear, introducing a *C*_P_′ differing from  eventually boils down to a postprocessing step, and does not influence the optimization itself. In other words, the actual results on power increase reported in this paper, which are all non-dimensionalized by a reference power, are not affected by the value of *a*.

## Appendix B. Derivation of adjoint forcing and cost functional gradient

In this appendix, we expand on the derivation of the adjoint forcing terms and the cost functional gradient in equations (2.12) and (2.13), respectively. For the derivation of the standard adjoint transport terms in the Navier–Stokes equations, we refer the reader elsewhere [13,14,31]. The derivation of the adjoint formulations of the wall stress model and Smagorinsky model is elaborated by Goit & Meyers [3]. Following the same approach as the latter study, we construct the Lagrangian of the optimization problem by adding the state constraints, with shorthand notation ***B***(***φ***,***q***), to the cost functional  through an inner product with a set of Lagrange multipliers ***q****=[***ξ***,*π*,***σ***] to form

B 1

B 2

The adjoint equations in (2.9)–(2.11) can then be found by expressing  for each of the state variables ***q***_*i*_.

**(a) Adjoint of time-filtered thrust coefficient**

We find the adjoint time filter equation by expressing the Riesz representation of . Using equations (B 2), (2.1), (2.6) and (2.7), and applying partial integration to the transient term, leads to

B 3

B 4

B 5

This equation directly leads to the adjoint time-filter equation (2.11). The boundary term introduced by partial integration vanishes by imposing the terminal condition *σ*_*k*_(*T*)=0. The factor *a* originates from the relationship between  and *C*_P_′ derived in appendix A.

**(b) Adjoint forcing term**

Similar to the derivation of the corresponding term in [3], we find the adjoint forcing term through

B 6

B 7

B 8

B 9

leading to equation (2.12).

**(c) Gradient**

The gradient of the reduced cost functional  with respect to the control variables ***φ***≡[*C*′_T,1_,…,*C*′_T,*N*_*t*__] is derived from (B 2) as

B 10

or , with ***σ***≡[*σ*_1_, …, *σ*_*N*_*t*__].

## Appendix C. Variance estimation of energy gains

We can estimate the standard deviation of *E*_O_/*E*_*R*_ based on *N* optimization windows, where *N*=14, including all windows except the first, which exhibits transient behaviour for the optimized cases (figure 10). Because the integral time scale of power extraction *Λ*≈50 s is significantly smaller than the window time horizon *T*_*A*_=120 s, time-integrated quantities over different windows can be assumed to be statistically independent. First, the total time-averaged power is defined as

C 1

hence . Furthermore, the window-averaged power extraction for each optimization window *i* is

C 2

Using a Taylor series approximation for the ratio , the variance thereof can be expressed in terms of statistical quantities of its numerator and denominator (e.g. [32]) as

C 3

where var, cov and E denote the variance, covariance and expected values respectively. Since , the variances in equation (C 3) can be written as . Furthermore, combining properties of the covariance operator with the fact that mean powers in different optimization windows can be assumed to be statistically independent, the covariance in equation (C 3) can be rewritten as

C 4

C 5

C 6

C 7

Finally, since , equation (C 3) can be readily evaluated in terms of quantities that can either be directly obtained from total time-averaged powers  and , or calculated from the distribution of window-averaged powers  and  as

C 8

leading to a standard deviation . Note that, even though  and  are normally distributed, the ratio between them is not. Therefore, the interval of , as shown in the error bars in figure 8, does not correspond to a 95% confidence interval as would be the case for a normal distribution of . Nonetheless, by definition of the standard deviation, the  interval provides a good uncertainty estimate for the gains reported in figure 8.
